# Supplementary material for: Comprehensive Annotation and Functional Exploration of MicroRNAs in Lettuce
Source: Front Plant Sci. 2021 Dec 24;12:781836. doi: 10.3389/fpls.2021.781836 (PMC8739914; doi:10.3389/fpls.2021.781836)
Supplement: Supplementary file 2 [file Data_Sheet_8.ZIP › GSM2230750.plot/Lsa-miR408_Lsat_1_v5_gn_3_92421.1_245_TPlot.pdf]

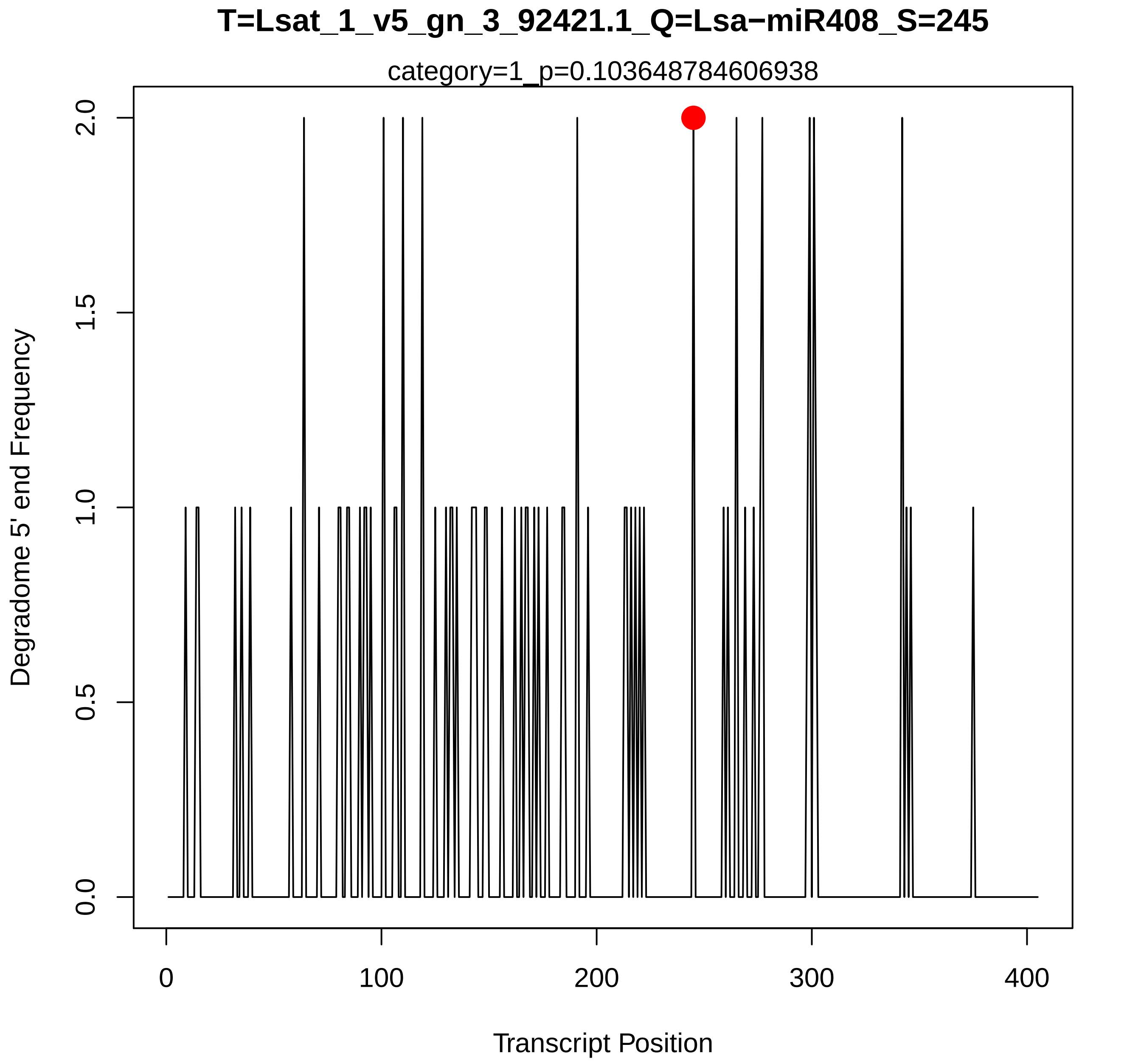
T=Lsat_1_v5_gn_3_92421.1_Q=Lsa−miR408_S=245
category=1_p=0.103648784606938
Degradome 5' end Frequency
0.0
0.5
1.0
1.5
2.0
0
100
200
300
400
Transcript Position
